# Oral Microbiome Profiles and Inflammation in Pregnant Women Who Used Orthodontic Appliances

**DOI:** 10.3390/dj10070118

**Published:** 2022-07-01

**Authors:** Fajar Kusuma Dwi Kurniawan, Retno Indrawati Roestamadji, Nobuhiro Takahashi, Udijanto Tedjosasongko, Ida Bagus Narmada, Meircurius Dwi Condro Surboyo, Indeswati Diyatri

**Affiliations:** 1 Faculty of Dental Medicine, Universitas Airlangga, Surabaya 60132, Indonesia; kurifana@gmail.com; 2 Department of Oral Biology, Faculty of Dental Medicine, Universitas Airlangga, Surabaya 60132, Indonesia; indeswati-d@fkg.unair.ac.id; 3 Graduate School of Dentistry, Tohoku University, Sendai 980-8575, Japan; nobuhiro.takahashi.a5@tohoku.ac.jp; 4 Department of Pediatric Dentistry, Faculty of Dental Medicine, Universitas Airlangga, Surabaya 60132, Indonesia; udijanto@fkg.unair.ac.id; 5 Department of Orthodontics, Faculty of Dental Medicine, Universitas Airlangga, Surabaya 60132, Indonesia; ida-b-n@fkg.unair.ac.id; 6 Department of Oral Medicine, Faculty of Dental Medicine, Universitas Airlangga, Surabaya 60132, Indonesia; meircurius@fkg.unair.ac.id

**Keywords:** fixed orthodontic, microbiome, orthodontics, pregnancy, IL-6, inflammation, TNF-α

## Abstract

It is common for women to undergo orthodontic treatment during pregnancy, especially through the use of fixed orthodontic devices. In changing the oral microbiome profile, it is crucial to increase the immune responses of pregnant women using fixed orthodontics; however, changes in the microbiomes of pregnant women with orthodontic appliances can be adjusted. Therefore, we aimed to conduct research on the oral cavity microbiome profiles, specifically IL-6 and TNF-α, of pregnant women using fixed orthodontic appliances. We proposed an observational analysis of 30 third-trimester pregnant women. OHI-S was recorded, saliva collection was performed using the passive drool method for IL-6 and TNF-α, and analysis and mucosal swabs were used to determine the oral microbiome profile. Kruskal–Wallis and post hoc Bonferroni tests were used to identify any significant differences with values of *p* < 0.05. Of these pregnant women, those with orthodontic appliances developed 10 types of bacteria at similar levels (>80%) from the genera *Streptococcus*, *Lactobacillus*, and *Veillonella*. There was no difference between the oral microbiomes of the control group and the pregnant women with a history of orthodontic appliance use. While the level of TNF-α in the women with orthodontic appliances was higher compared with the control group who had never used orthodontic appliances (*p* < 0.05), there was no difference in the IL-6 levels. The IL-6 and microbiome profile produced normal results, so the use of orthodontic appliances during pregnancy should be allowed with conditions. Pregnant women with orthodontic appliances must keep the oral cavity clean and their appliances well-maintained to avoid oral problems.

## 1. Introduction

Research on the diversity of human microbiomes was initiated by Antonie van Leewenhoek in the early 1680s when he compared the microbiota of the oral cavity and faeces [1]. It is estimated that 96% of the bacteria found in the microbiomes of the oral cavity and intestines are similar, including the phyla *Firmicutes*, *Bacteroidetes*, *Proteobacteria*, *Actinobacteria*, *Spirochaetes*, and *Fusobacteria* [2].

When the body undergoes physiological and even pathological changes, the microbiome of the body also changes [3]. During pregnancy, women experience changes in their intestinal microbiome profile [4]. These changes can be affected by nutrition and may alter foetal health [5]. However, this does not only occur in the intestines; microbiome changes also occur in the oral cavity. In the first and third trimesters of pregnancy, it was found that the numbers of *Porphyromonas gingivalis* and *Aggregatibacter actinomycetemcomitans* increased significantly compared to non-pregnant women [6,7]. In addition to these two bacteria, *Candida albicans* also increased during the second and third trimesters of pregnancy. *Streptococcus mutans* showed a similar increase but was not quite as dramatic in the first trimester and remained constant until the third trimester [8].

Oral hygiene is necessary to prevent caries and periodontal disease during and after a fixed orthodontic treatment. Instruction in oral hygiene is essential to all cases of orthodontic treatment and must be reinforced [9]; pregnant women are no exception. During three months of fixed orthodontic bonding, there was a significant increase in the number of *Porphyromonas gingivalis*, *Tannerella forsythia*, *Porphyromonas intermedia*, *Porphyromonas nigrescens*, and *Fusobacterium* spp., which were previously found in the periodontal ligament [10]. Other bacteria such as *Enterobacter cloacae*, *Klebsiella oxytoca*, *Klebsiella pneumoniae*, and *Serratia marcescens* emerged, even in people without orthodontic devices [11].

IL-6 is a multifunctional cytokine that plays an important role in acute and chronic inflammation and autoimmunity. In relation to orthodontic treatment, IL-6 plays an important role in the movement of teeth, namely the inflammatory process that supports the resorptions process in apical areas [12,13]. Increased IL-6 levels occur not only in periodontal or alveolar tissues but also in saliva [14]. Similar to IL-6, TNF-α is also affected by orthodontic treatment and plays a role in the processes of tooth movement and bone resorption [15]. Orthodontic treatment increases TNF-α levels in periodontal tissue, especially in gingival crevicular fluid [16]. With the inclusion of various inflammatory markers such as cytokines, namely TNF-α and IL-6, it also affects the hygiene and oral composition of the microbiome [17].

This current study aimed to identify the composition of bacteria in the microbiome, derived from mucosal swabs, and the levels of TNF-α and IL-6 found in saliva. Orthodontic treatments trigger changes in the microbiome profile that may cause abnormalities in both the soft and hard tissues of the oral cavity. During orthodontic treatment and pregnancy, there is a significant increase in oral microbiome profile changes, which can lead to various diseases of the oral cavity. Pregnancy naturally results in a wide range of tissue abnormalities. This includes the dental tissues and could affect oral health.

The hypotheses were as follows:

1. That the use of orthodontic appliances in pregnant women changes the microbiome profile.
2. That the levels of TNF-α and IL-6 found in saliva are higher in pregnant women that have used orthodontic appliances.

## 2. Materials and Methods

### 2.1. Study Participant

This research was an observational analytical study. The participants in this study were all pregnant women in their third trimester who visited Kendangsari Mothers and Child Hospital in Surabaya. The inclusion criteria for this research were pregnant women in their third trimester with no caries or periodontal disease. The exclusion criteria were pregnant women who take medicine that may affect oral cavity homeostasis or the presence of systemic disease.

A sample of 32 participants was selected and divided into 3 groups. The control group was pregnant women who had never used orthodontic appliances, the second group was pregnant women who had previously used orthodontic appliances, and the third group was pregnant women with current orthodontic appliances.

### 2.2. Oral Health Assessment

Subjects were instructed to sit on normal chairs and clinical examinations were performed. The oral hygiene index (OHI-S) is the sum of the debris index (DI) and calculus index (CI) [18]. DI and CI examinations were carried out using a periodontal probe (Probe UNC15, Osung, Osung MND Co LTD, Gyeonggi-do, Korea) placed on the tooth surface horizontally. To obtain the DI score, the sum of the debris assessment was divided by the number of teeth examined. The sum of the calculus scores was then divided by the number of teeth examined to obtain a CI score. These scores were divided into good OHI-S (0–1.2) and fair OHI-S (>1.3).

### 2.3. Saliva Collection

Saliva collection was performed using the passive drool method, which involved lowering of the head and releasing saliva collected at the base of the mouth into a 15 mL Eppendorf tube. Subjects were instructed not to eat or drink for about one hour before the salivary sample collection. They were asked to gargle with water for approximately one minute, and then wait five minutes [19]. Salivary samples were collected up to 5 mL [19], and placed in a refrigerator at a temperature of −30 °C.

### 2.4. Mucosal Swab

Subjects were instructed not to eat or drink for one hour before the procedure. The entire surface of the oral cavity was rubbed using a sterile cotton swab, and the swab then was placed in a phosphate buffer saline solution. The mucosal swab samples were kept at −80 °C until testing was carried out.

### 2.5. DNA Extraction

DNA extraction of samples was carried out in accordance with the factory protocol of the kit used (QIAamp DNA Stool Mini Kit—Qiagen, Hilden, Germany). The sample was taken from the Eppendorf tube and placed in a microcentrifuge tube, and then 180 μL Buffer ATL and 20 μL proteinase K were added to the same tube. The tube was vortexed for 15 s and then increated at 600 °C for 24 h. Following this, 200 μL Buffer AL was added and the tube was vortexed for 15 s, and then increated for 10 min at 70 °C. Finally, 200 μL 96% ethanol was added, vortexed for 15 s, and then placed in spin down. The mixed results were placed into the QIAamp Mini spin column and centrifuged at a speed of 8000 rpm for 1 min.

The concentration and quality of DNA was determined using a Nano-Drop 1000 spectrophotometer (Thermo Fisher Scientific, Wilmington, DE, USA). At the end of the extraction, 50 μL of DNA sample was obtained. This DNA was used for the qPCR running process.

### 2.6. qPCR for Microbiome Analysis

PCR Master mix (Intron; iNtRON Biotechnology, Gyeonggi-do, Korea), Universal Forward Primer Macrogen (5′-GAG AGT TTG ATY CTG GCT CAG-3′), Universal Reverse Primer Macrogen (5′-GAA GGA GGT GWT CCA RCC GCA-3′), and DNA Marker 1 kbp (Thermo Fisher Scientific, Wilmington, DE, USA).

### 2.7. TNF-α and IL-6 Levels

The TNF-α and IL-6 levels were analysed using an ELISA kit. TNF-α is human tumour necrosis factor α (Bioassay Technology Laboratory, Shanghai, China) and IL-6 is human interleukin 6 (Bioassay Technology Laboratory, Shanghai, China).

### 2.8. Statistical Analysis

The levels of TNF-α and IL-6 were analysed with the Kolmogorov–Smirnov test to assess the data distribution and the Levene test for data homogeneity. Next, the Kruskal–Wallis and post hoc Bonferroni tests were used to discover any significant differences with values of *p* < 0.05. SPSS version 24 (IBM SPSS Statistic 24 for mac, New York, NY, USA) was used for the analysis.

## 3. Results

### 3.1. Subject Characteristics

**Figure 1:**
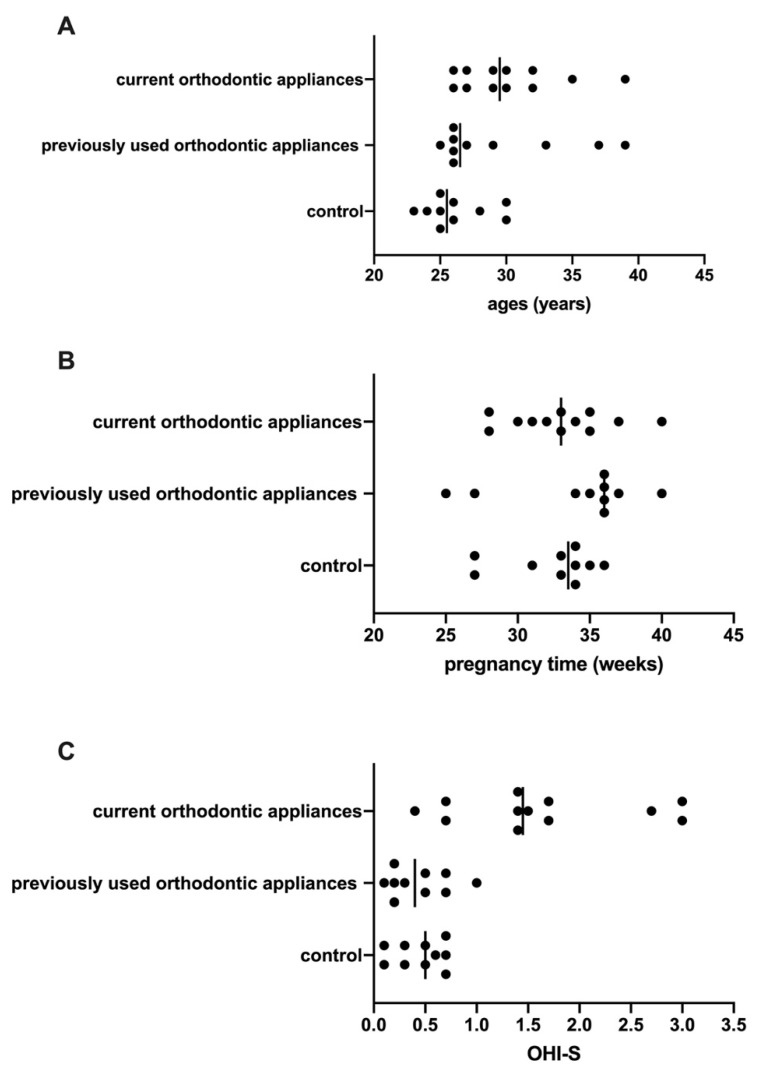
Demographic information. Ages (**A**); week of pregnancy (**B**); and OHI-S (**C**).

The pregnant women who had never used orthodontic appliances (control) had an average age of 26.2 ± 2.4 years old, the women who had previously used orthodontic appliances were, on average, 29.4 ± 5.1 years old, and those with current orthodontic appliances were, on average, 30.6 ± 3.9 years old (Figure 1A).

The average stage of pregnancy for women who had never used orthodontic appliances (control) was 32.4 ± 3.2 weeks; for those who had previously used orthodontic appliances, it was 34.2 ± 4.6 weeks; and for those with current orthodontic appliances, it was 33.0 ± 3.5 weeks (Figure 1B).

### 3.2. OHI-S Status

For OHI-S, the pregnant women who had never used orthodontic appliances (control) had an average score of 0.5, the women who had previously used orthodontic appliances had an average score of 0.4, and those with current orthodontic appliances had an average score of 1.6 (Figure 1C).

### 3.3. qPCR for Microbiome Analysis

**Figure 2:**
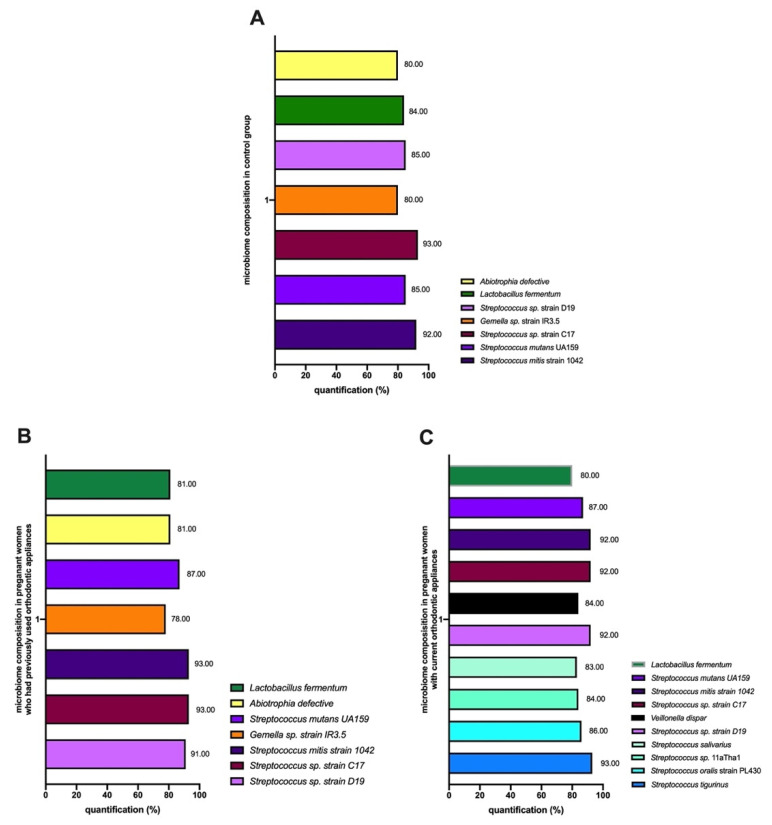
qPCR results of the similarities in the bacteria in the oral microbiomes of pregnant women who had never used orthodontic appliances (control) (**A**); pregnant women who had previously used orthodontic appliances (**B**); and pregnant women with current orthodontic appliances (**C**).

The qPCR results showed that the microbiome in pregnant women who had never used orthodontic appliances (control) consisted of seven types of bacteria with similar levels (>80%) from the genera *Streptococcus*, *Gemella*, *Lactobacillus*, and *Abiotrophia* (Figure 2A). The oral bacteria found in the control included *Streptococcus mitis* strain 1042, *Streptococcus mutants* UA159, *Streptococcus* sp. strain C17, *Streptococcus* sp. strain D19, *Gemella* strain IR3.5, *Lactobacillus fermentum*, and *Abiotrophia defective*.

The microbiome in pregnant women who had previously used orthodontic appliances consisted of seven types of bacteria with similar levels (>80%) from the genera *Streptococcus*, *Gemella*, *Lactobacillus*, and *Abiotrophia*; no differences were observed with the microbiome of pregnant women who had never used orthodontic appliances (Figure 2B). The oral bacteria consisted of *Streptococcus mitis* strain 1042, *Streptococcus* sp. strain C17, *Streptococcus* sp. strain D19, *Streptococcus mutants* UA159, *Gemella* strain IR3.5, *Lactobacillus fermentum*, and *Abiotrophia defective*.

In the pregnant women with current orthodontic appliances, there were 10 types of bacteria with similar levels (>80%) from the genera *Streptococcus*, *Lactobacillus*, and *Veillonella* (Figure 2C). The oral bacteria were *Streptococcus tigurinus*, *Streptococcus mitis* strain 1042, *Streptococcus oralis* strain PL430, *Streptococcus* sp. *11aTha1*, *Streptococcus salivarius*, *Streptococcus* sp. strain D19, *Streptococcus* sp. strain C17, *Streptococcus mutants* UA159, *Lactobacillus fermentum*, and *Vellonelia dispar*.

### 3.4. TNF-α and IL-6 Value

**Figure 3:**
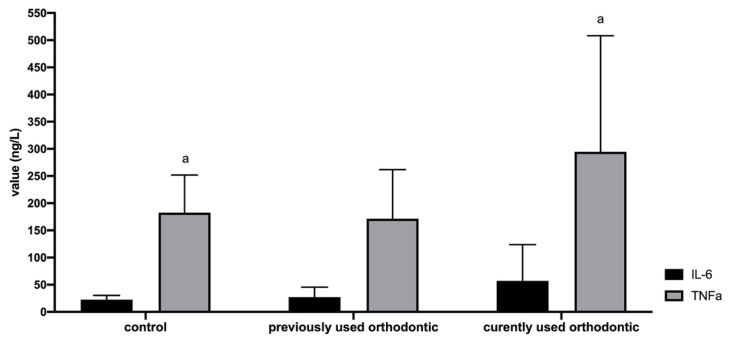
Values of salivary TNF-α and IL-6. ^a^Significant differences were shown using Kruskal-Wallis and post hoc Bonferroni tests with a significance value of *p* < 0.05.

The IL-6 levels in all three sample groups are shown in Table 1 and no significant differences were observed (Figure 3). The level of TNF-α in the pregnant women with current orthodontic appliances was higher compared with pregnant women who had never used orthodontic appliances (control) (*p* < 0.05) while the level of TNF-α in pregnant women with a history of orthodontic appliances showed no significant difference from those with current orthodontic appliances (Figure 3).

## 4. Discussion

The analysed data showed similarity in the oral microbiomes. The control group and pregnant women who had previously used orthodontic appliances had similar oral microbiomes consisting of *Streptococcus mitis* strain 1042, *Streptococcus mutants* UA159, *Streptococcus* sp. strain C17, *Streptococcus* sp. strain D19, *Gemella* strain IR3.5, *Lactobacillus fermentum*, and *Abiotrophia defective*. The pregnant women with current orthodontic appliances had oral microbiomes with five similar bacteria but did not include the *Gemella* strain IR3.5. Other bacteria also found included *Streptococcus tigurinus*, *Streptococcus oralis* strain *PL430*, *Streptococcus* sp. *11aTha1*, *Streptococcus salivarius*, and *Vellonelia dispar*. The results of this study show that the microbiomes of all the groups were dominated by *Streptococcus* strain. These bacteria are the dominant species of the oral microbiome [20].

In pregnant women who had previously and currently had fixed orthodontic appliances, it was found that the most dominant bacteria were *Streptococcus mitis* strain 1042, *Streptococcus mutants* UA159, *Streptococcus* sp. strain C17, *Streptococcus* sp. strain D19, and *Lactobacillus fermentum*. *Streptococcus mitis* is a type of viridians streptococcus and commensal normal flora of the oral cavity. *Streptococcus mitis* is a bacterium that initiates colonisation in the human oral cavity and oropharynx. It can move from its normal habitat and cause many complications such as endocarditis, bacteraemia, and septicaemia. This bacterium is an opportunistic pathogen [21]. The presence of *Streptococcus* sp., especially *Streptococcus mutants*, was reported to increase with the placement of an orthodontic appliance. The appliance becomes an iatrogenic factor that increases the risk of plaque and biofilm formation on the teeth and the appliance itself [22]. This accumulation can lead to disease of the teeth, gingiva, and periodontium. The current study confirmed that the oral microbiome is dominated by *Streptococcus* strain in pregnant women without orthodontic appliances, with a history of orthodontic appliances, and with current orthodontic appliances. Knowledge of the microbiome is very important as it has been demonstrated that the use of probiotics [23] and natural compounds [24] can modify clinical and microbiological parameters in periodontitis patients. The use of these products could also have an effect in pregnancy, and these variables should be considered in future clinical trials [25].

The dominance of the *Streptococcus* strain was observed among both pregnant women and non-pregnant women. Not only did the species change but the number of microorganisms also increased in pregnant women [26]. In non-pregnant women, the *Streptococcus* strain was dominated by *Streptococcus agalactiae* [27]. For pregnant women, the *Streptococcus mutants* increased compared with non-pregnant women [28]. In the present study, *Streptococcus mutants* was also found in pregnant women who had never used orthodontic appliances. The changes in these bacteria [29] are related to increased levels of oxidative stress such as malondialdehyde (MDA) [28], and cause a decrease in the salivary acidity and calcium content [30]. Due to this condition, the risk of dental caries and gingival disease increases. These findings show that oral cavity health in pregnant women must be considered, even down to the decision of using or not using orthodontic appliances.

Increased oxidative stress also creates an increase in the inflammatory response in the oral cavity. Various abnormalities in the soft tissues of the oral cavity of pregnant women include gingivitis and periodontitis. These diseases lead to an increase in the salivary IL-6 and TNF-α levels of pregnant women compared with pregnant women without gingivitis and periodontitis [31]. In this research, the IL-6 levels of all three sample groups showed no significant differences. IL-6 has a role in regulating paracrine to maintain foetal growth and placental hormone production. IL-6 also contributes to the development of the immune and hematopoietic system of the foetus [32,33].

The level of TNF-α in pregnant women with orthodontic appliances was higher compared with pregnant women who had never used orthodontic appliances. Physiological changes during pregnancy lead to changes in the oral mucosa. Pregnant women commonly experience diseases such as caries and periodontitis. Periodontitis causes an increase in the salivary-soluble TNF-α receptor, which leads to an increase in the TNF-α level [34]. An increase in the salivary TNF-α level in pregnant women, especially those who are obese, can affect the weight of the infant [35]. On the other hand, tooth movement caused by orthodontic force may increase salivary TNF-α [36] and IL-6 levels [14]. It can be concluded that, in the absence of pregnancy, the concentration of salivary IL-6 and TNF-α increases due to orthodontic appliance use. The risk of inflammation in pregnant women with orthodontic appliances is also caused by high OHI-S. Orthodontic appliance use can also increase plaque formation, which may impact the health of the gingiva and periodontium [37]. Oral hygiene is important to maintain and even increase, especially in pregnant women with orthodontic appliances, in order to prevent caries, gingivitis, and periodontitis. To maintain oral hygiene, several strategies are needed, including daily use of fluoride toothpaste and mouthwash. Daily use of both shows a reduced risk of caries during orthodontic treatment [38].

It should be noted that in orthodontics, over the past few years, research on the use of wire orthodontics derived from NiTi (nickel-titanium) on the adhesion of *Streptococcus mutants* has been performed. Various concentrations of Ni in the orthodontic wire influence the adhesion of *Streptococcus mutants*. Research by Pavlic et al. showed that Ni concentrations of 1000 ug/mL significantly inhibit adhesion [39]. Other research on the prevention of bacteria adhesion on orthodontic wire by applying titanium dioxide has been carried out [40,41]. The relationship with the maintenance of oral hygiene and daily use of fluoride toothpaste is also reported have an influence on the release of Ni [42,43], so it is likely that it also has an influence on the adhesion and formation of biofilms on orthodontic appliances.

## 5. Conclusions

The conclusion of this study is that the microbiome profile and IL-6 value showed normal results, so the use of orthodontic appliances during pregnancy should be allowed under certain conditions. Pregnant women who have orthodontic appliances must keep the oral cavity in good condition and maintain their orthodontic appliances periodically to avoid oral cavity problems. The limitation of this study is the short observation time. Future research should be conducted in a study encompassing multiple centres to strengthen this result.

## Figures and Tables

**Table 1:** Values of salivary TNF-α and IL-6 in each group.

Groups	IL-6 Value (ng/L)			TNF-α Value (ng/L)		
	Mean ± SD	Minimum	Maximum	Mean ± SD	Minimum	Maximum
pregnant women who had never used orthodontic appliances (control)	22.38 ± 7.94	13.53	40.74	182.57 ± 69.24	84.55	294.22
pregnant women who had previously used orthodontic appliances	27.23 ± 18.22	9.78	75.69	171.41 ± 90.50	77.76	333.18
pregnant women with current orthodontic appliances	57.13 ± 66.71	17.13	260.46	294.78 ± 213.35	81.60	909.38

SD: standard deviation.

## Data Availability

The datasets used and analysed during the current study are available from the corresponding author upon reasonable request.
